# Functional versus conventional strength and conditioning programs for back injury prevention in emergency responders

**DOI:** 10.3389/fbioe.2022.918315

**Published:** 2022-09-09

**Authors:** Pui Wah Kong, Tommy Yew Weng Kan, Roslan Abdul Ghani Bin Mohamed Jamil, Wei Peng Teo, Jing Wen Pan, Md Noor Hafiz Abd Halim, Hasan Kuddoos Abu Bakar Maricar, David Hostler

**Affiliations:** ^1^ Physical Education and Sports Science Academic Group, National Institute of Education, Nanyang Technological University, Singapore, Singapore; ^2^ Responder Performance Centre, Civil Defence Academy, Singapore Civil Defence Force, Singapore, Singapore; ^3^ Center for Research and Education in Special Environments, University at Buffalo, Buffalo, CA, United States

**Keywords:** firefighters, paramedics, stiffness, EMG, fatigability, low back pain, Oswestry Disability Index, isometric strength

## Abstract

Back pain and back-related injuries are common complaints among emergency responders. The purpose of this study was to compare the effectiveness of two strength and conditioning programs in improving back muscle characteristics and disabilities in emergency responders (firefighters/paramedics). Participants (*n* = 24) were randomized into two groups to complete 16 weeks of supervised exercise intervention: 1) Functional training used unilateral movements that mimicked the asymmetrical nature of emergency operations, 2) Conventional training performed bilaterally loaded exercises. Outcome measures were maximum isometric back extension strength, passive muscle stiffness, lumbar extensor fatigability, and revised Oswestry Low Back Pain Questionnaire. A mixed model Analysis of Variance with repeated measures was performed to compare the difference over time and between groups. While the training effects were similar between groups, both programs improved isometric back extension strength (+21.3% functional, +20.3% conventional, *p* < 0.001, η_p_^2^ = 0.625) and lumbar extensor muscle fatigability (+17.4% functional, +9.5% conventional, *p* = 0.009, η_p_^2^ = 0.191). Bilateral symmetry in muscle stiffness was improved as indicated by reduction in symmetry index (-7.1% functional, -11.8% conventional, *p* = 0.027, η_p_^2^ = 0.151). All self-reported pain and disability scores fell within the category of “*minimum functional limitation*” throughout the intervention and 6-month follow-up periods. For frontline firefighters and paramedics, both functional and conventional strength training are effective for improving back muscle characteristics.

## 1 Introduction

Emergency response plays a critical role in ensuring public safety, but it is an inherently dangerous occupation. Emergency situations warranting a response can range from natural disasters to home fire and transportation incidents. Emergency responders such as firefighters and paramedics must be physically fit in order to with the challenges during emergency situations (Beach et al., 2014). Considering the exposures to strenuous and physically demanding tasks (Nazari et al., 2020b; Stassin et al., 2021), it is not surprising to note frequent complaints of low back pain (LBP) and back injuries among emergency responders. For instance, the prevalence of LBP in firefighters has been reported to be approximately 30% across many countries (Katsavouni et al., 2014; Negm et al., 2017; Damrongsak et al., 2018; Nazari et al., 2020a; Pelozato de Oliveira et al., 2021) and up to 71.1% in South Korea (Kim and Ahn, 2021). A similar range from 31.8 to 85.1% of LBP prevalence was also observed in emergency medical services (EMS) personnel (Ham and Ahn, 2008; Kim et al., 2017; Aljerian et al., 2018; Imani et al., 2018). In additional to the dangerous nature of emergency work, inadequate level of physical fitness may also increase the risk of work-related injuries (Griffin et al., 2016). One possible strategy to reduce back pain and injuries in emergency responders is to design and implement an effective strength and conditioning program to improve back muscle strength and overall fitness (Abel et al., 2015).

Resistance training is a key element in all well-rounded strength and conditioning programs. Traditionally, conventional resistance training emphasizes symmetrical exercises whereby the left and right limbs are loaded together and perform the same range of motion simultaneously (bench press, back squat, deadlift, strict press) (Beach et al., 2014; Moon et al., 2015). These conventional exercises, when performed at appropriate intensity and volume, may provide sufficient back and core strength to protect the emergency responders from back injuries. In emergency work, however, the operational tasks such as swinging an axe, advancing hose, and handling casualties are not symmetrically loaded. Thus, functional exercises, defined here as loading of a single limb and activating the core from shoulder to opposite hip, better simulate the asymmetrical nature of the emergency response tasks. High-intensity functional training programs have been shown to be safe and effective for the military and general population (Poston et al., 2016; Feito et al., 2018). While both conventional and functional fitness programs should target all major muscle groups, a functional program that takes firefighting and paramedic related tasks into consideration may be superior to improve back muscle characteristics and thereby reducing back pain and injuries.

The purpose of study was to compare the effectiveness of two strength and conditioning programs on back muscle characteristics and disabilities in emergency responders in Singapore. It was hypothesized that functional training would be more effective than conventional training in improving back muscle strength, stiffness, fatigability, and self-report disability outcomes. This study will pioneer the implementation of customized strength and conditioning programs among frontline emergency responders at their workplace. Findings from this study can provide empirical evidence on whether such physical training programs can improve back muscle function and disabilities. Such information can guide future practice and policy pertaining to workplace safety and well-being of emergency responders.

## 2 Materials and methods

### 2.1 Study design

**FIGURE 1:**
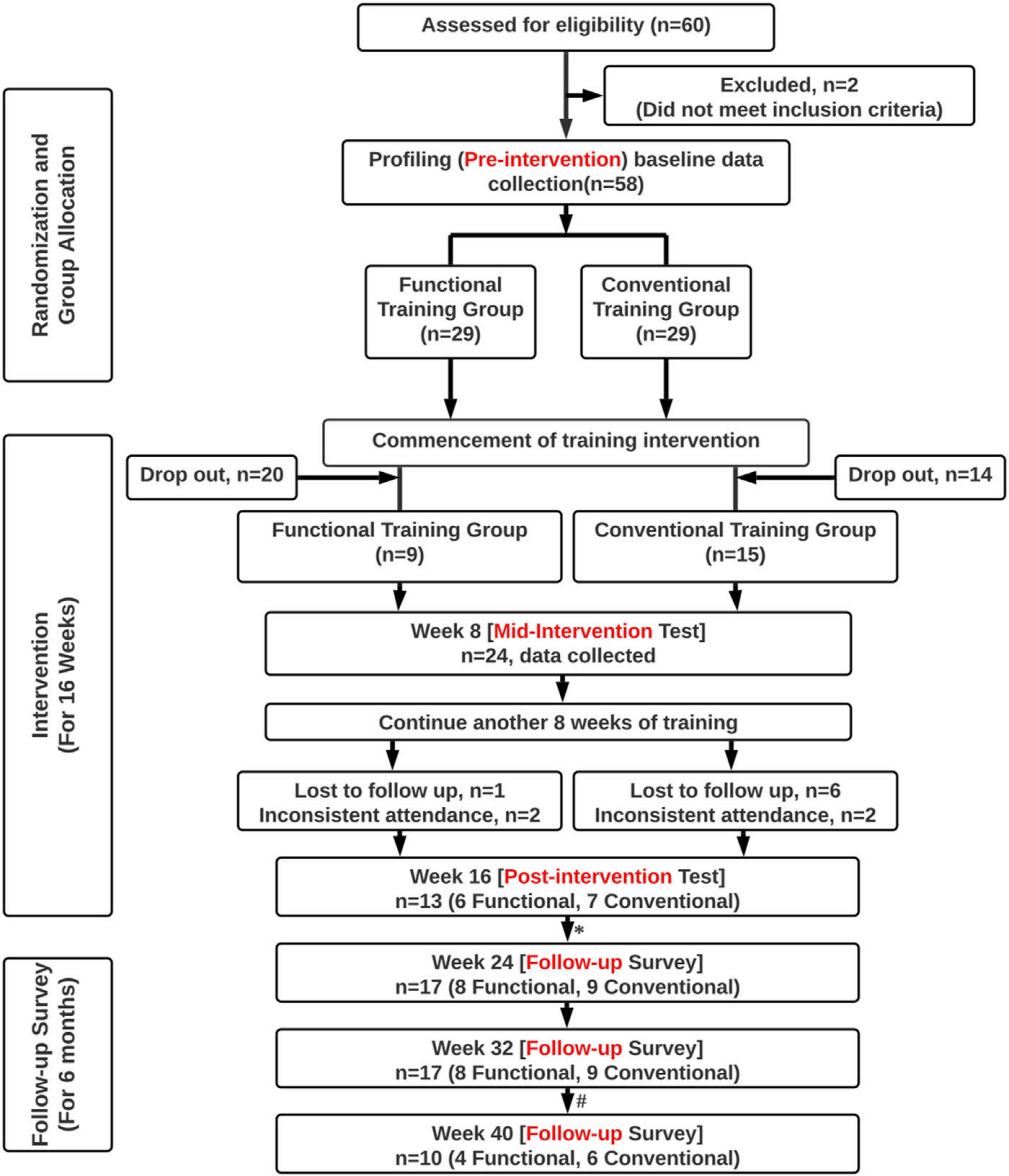
Study overview. [Note. *4 participants (2 Functional, 2 Conventional) who did not attend the physical post-intervention test at Week 16 were able to complete the online follow-up survey at Week 24. # 7 participants (4 Functional, 3 Conventional) were lost to contact at Week 40.].

This was a longitudinal training study with two arms (Figure 1). Participants were randomly allocated into one of the two exercise groups undergoing different types of strength and conditioning programs for 16 weeks. The Functional Group performed exercises that simulated the asymmetrical and/or diagonal loading nature of the movements which emergency responders would encounter in their regular work routine. The Conventional Group was prescribed traditional strength training that comprised bilaterally symmetrical multi-muscle group exercises. Back muscle characteristics were assessed at pre-intervention (baseline), mid-intervention, and post-intervention. Self-report pain and disability surveys were administered as pre-intervention, mid-intervention, post-intervention, and follow-ups at 2, 4, and 6 months after the intervention.

### 2.2 Participants

This study was approved by the Nanyang Technological University Institutional Review Board (Protocol Number: IRB-2020-06-85). All participants provided written informed consent to enroll in the study. The inclusion criteria were that participants were 1) males or females, 2) between the age of 21 and 45 years old, 3) a full-time emergency responder at the Singapore Civil Defence Force (SCDF), and 4) healthy to perform work duties at the time of the recruitment. Participants were excluded if they had any histories of back surgery or self-reported to be pregnant (females only).

At the start of the study, a total of SCDF 58 emergency responders who met the inclusion criteria have enrolled. Due to the unforeseeable circumstances associated with COVID-19, we encountered a high drop-out rate in the early phase of the study, shortly after the pre-intervention test. Since the early drop-outs did not engage in sufficient exercise training to allow meaningful data analysis, we adopted the modified intention-to-treat approach (Gupta, 2011; Montedori et al., 2011). Only participants who had completed at least 8 weeks of training and the mid-intervention test were included in the data analysis. The physical characteristics of these 24 male emergency responders (23 firefighters, 1 administration officer) are shown in Table 1.

**TABLE 1:** Physical characteristics and demographic background of emergency responders (*n* = 24).

Characteristics	All 24	Functional Group (8 firefighters, 1 officer)	Conventional Group (15 firefighters)	*P*
Ethnicity	Malay (*n* = 18)	Malay (*n* = 7)	Malay (*n* = 12)	--
	Chinese (*n* = 3)	Chinese (*n* = 2)	Chinese (*n* = 1)	
	Others (*n* = 3)	Others (*n* = 1)	Others (*n* = 2)	
Age (years)	32.4 (5.2)	31.9 (5.0)	32.7 (5.4)	0.729
Body mass (kg)	73.4 (10.0)	70.1 (9.6)	75.6 (10.0)	0.193
Height (cm)	172.0 (7.5)	169.2 (8.4)	173.6 (6.8)	0.168
BMI (kg/m^2^)	24.8 (2.5)	24.5 (3.0)	25.0 (2.3)	0.618
LBP history	Yes (*n* = 13)	Yes (*n* = 7)	Yes (*n* = 6)	--
	No (*n* = 11)	No (*n* = 2)	No (*n* = 9)	
Pain disability	Minimum (*n* = 22)	Minimum (*n* = 7)	Minimum (*n* = 15)	--
	Moderate (*n* = 2)	Moderate (*n* = 2)	Moderate (*n* = 0)	
Regular smoker	Yes (*n* = 6)	Yes (*n* = 1)	Yes (*n* = 5)	--
	No (*n* = 18)	No (*n* = 8)	No (*n* = 10)	
Alcohol consumer	Yes (*n* = 1)	Yes (*n* = 1)	Yes (*n* = 0)	--
	No (*n* = 23)	No (*n* = 8)	No (*n* = 15)	

All 24 participants were males. Data are expressed as mean (SD) unless otherwise stated. Ethnicity (Others) comprises 1 Indian, 1 Javanese, and 1 Boyanese. BMI denotes body mass index. LBP denotes low back pain. Pain disability was measured with the Oswestry Disability Index (ODI). Differences between the Functional and Conventional groups were compared using independent t-tests.

### 2.3 Strength and conditioning exercise intervention

Participants were randomized into either the Functional Group or Conventional Group for exercise interventions. They were required to attend the exercise training on their duty days at their respective fire stations, two sessions per week for 16 weeks. All training was conducted in the gym at the assigned fire stations under the supervision of the same researcher (TYWK) who was trained and experienced in strength and conditioning.

**FIGURE 2:**
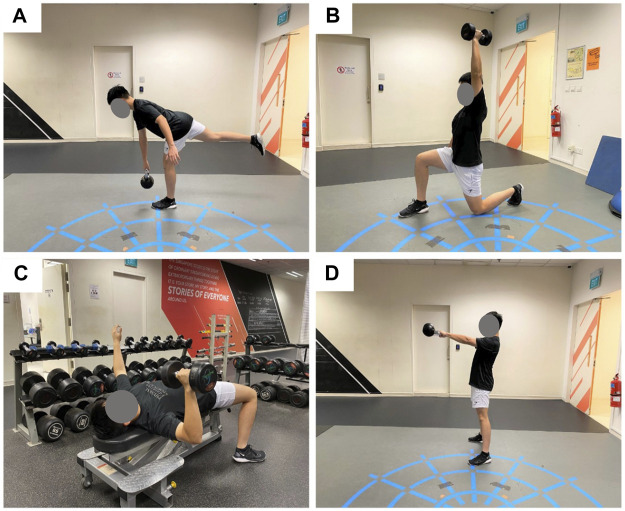
Example of Functional training exercises that are bilaterally asymmetrical and/or involve diagonal loading. **(A)** Single leg kettlebell deadlift. **(B)** Single arm dumbbell press. **(C)** Single arm chest press. **(D)** Kettlebell swing.

A typical training session consisted about 8 different types of exercises and lasted for 45–60 min (Table 2). In the beginning of each training session, participants from both groups went through the same sequence of warm-up exercises before the commencement of their specific training. Examples of warm-up exercises included 6 to 10 repetitions of forward lunges (with and without twist), dynamic chest stretch, single-leg hamstring stretch, walk-ins and T-spine rotation. All participants would execute four types of exercises: Exercises A, B, and C were designed to focus on different muscle groups while exercise D was largely cardiovascular-intensive. The Functional Group performed exercises that are bilaterally asymmetrical and/or involve diagonal loading (Figure 2). The Conventional Group was prescribed traditional strength training exercises that comprised bilaterally symmetrical multi-muscle group exercises (e.g., barbell back squat, push-up). Efforts were made to match the training load and training volume of the two groups as similar as possible. Detailed training programs for the full 16 weeks can be found in Supplementary Table S1 (Functional training) and Supplementary Table S2 (Conventional training).

**TABLE 2:** Sample exercises of functional group training program.

Session	Exercise	Repetition	Set
Week 1 Day 1	A1. DB Reverse Lunges	8—9 reps	3
	A2. DB Bench Press	8—9 reps	3
	B1. SL Kettlebell Deadlift	8—9 reps per leg	3
	B2. Bent Over Kettlebell Row	8—9 reps per arm	3
	C1. Forearm Plank	30 s	3
	C2. SA DB Row	8 reps	3
	D. Tabata	4 min	
	- Burpees	30 s	
	- Air Squats	30 s	
Week 1 Day 2	A1. DB Box Step Up	8—9 reps	3
	A2. Incline DB Bench Press	8—9 reps	3
	B1. Half Kneeling SA Press	8—9 reps	3
	B2. Bent Over Kettlebell Row	8—9 reps	3
	C1. Hollow Body Hold	20 s	3
	C2. Plank Hold DB Drag	8—9 reps	3
	D. EMOM	6 min	
	- 100 m Treadmill Run	60 s	
	- Bear Crawl Hold	60 s	

DB, denotes dumbbell; SL, denotes single leg; SA, denotes single arm; EMOM, denotes every minute on the minute.

Participants performed the prescribed exercises in groups with a superset training method where two exercises were done continuously with no long rest in-between. After completing one set of superset training (e.g., A1 and A2 in Table 2), the participants would rest for 2–3 min before commencing the following sets. Once the participants complete all the superset exercises (A, B and C), they would proceed to exercise D which was to perform either a Tabata training, EMOM (Every minute on the minute) training or AMRAP (As many rounds as possible) conditioning before ending the training session.

Participants were asked to indicate their rate of perceived exertion (RPE) of the exercise intensity after working out using the Borg’s CR-10 scale (Borg, 1990). On the very first day of training (i.e., Week 1 Day 1), participants of both groups were advised to work out at a RPE scale of 5 or 6 in a scale of 0 (nothing at all) to 10 (extremely strong). Subsequently, they were encouraged to progressively increase the intensity of training by either increasing the repetitions, weight or reducing the rest time between supersets as part of the progressive nature of the training program.

The training intervention was conducted in 2020–2021 during which we encountered great difficulties and uncertainties associated with COVID-19. For instance, there were restrictions and frequent updates of safe measures management (e.g., closure of gym facilities, limitation in group size, safe distancing measures). In addition to COVID-related measures, the work shift schedule of the emergency responders (e.g., 1 working day followed by 2 days off) also contributed to some interruptions in implementing the training intervention. Occasionally, the participants had to stop the exercise training to respond to an emergency immediately. These challenges required the research team to be flexible, adaptive, and ready for prompt action in order to execute the training study under sub-optimal conditions. For the participants’ safety, some adjustments to the number of repetitions, rest time and weights used were made for individual participants to accommodate absences from training.

### 2.4 Outcome measures

#### 2.4.1 Back extension strength

**FIGURE 3:**
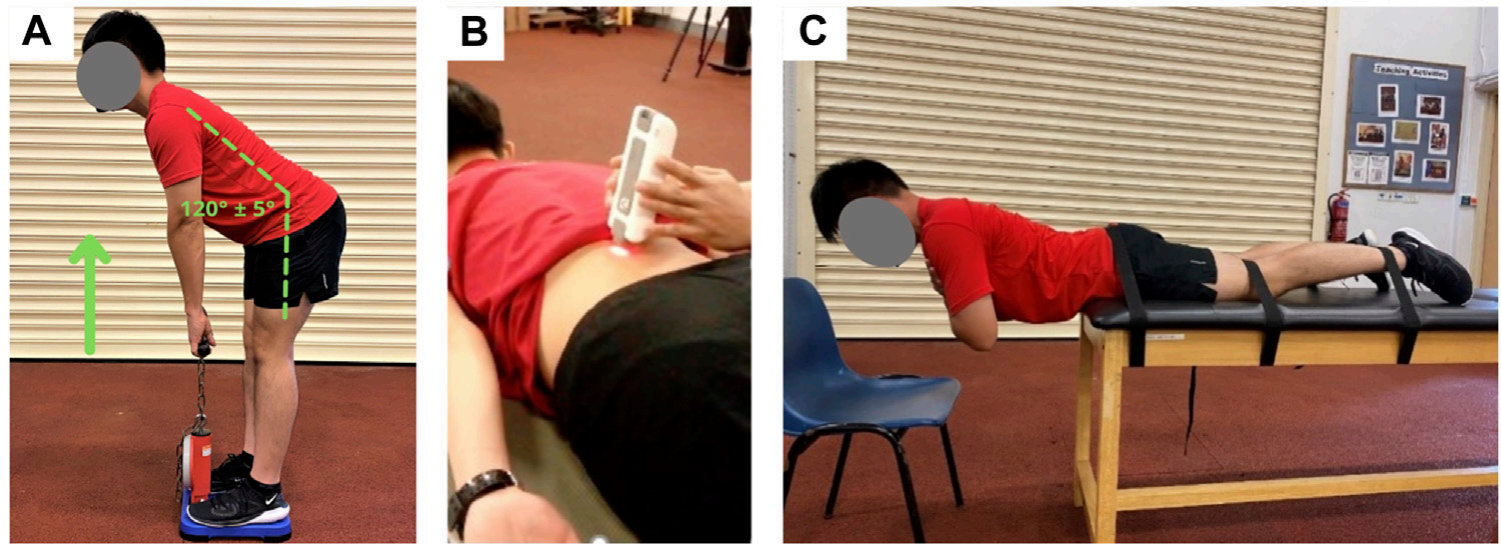
Back muscle characteristics assessment: **(A)** Maximal isometric hip extension test, **(B)** Passive stiffness of lumbar extensor muscles, **(C)** Modified Sorensen test for fatigability.

Maximal isometric back extension strength was measured using a dynamometer (Takei T.K.K.5402 BACK-D, Takei Scientiﬁc Instruments Co., Ltd, Tokyo, Japan) and expressed in kilograms (kg) of force produced. The hip angle was measured and standardized at 120 (±5) degrees before the commencement of the test (Figure 3A). This was to ensure that the testing postures and hence muscle lengths were similar across all participants and on different days. After a countdown “3, 2, 1, pull”, participants were asked to extend their back as hard as they could while holding on to the handle of the dynamometer for a minimum of 3 s. Participants were given a total of 3 attempts and the best maximum force produced was recorded. Sufficient time was given to rest between trials. The maximum force data were normalized to each participant’s body mass to facilitate comparison between groups. A normalized strength of 1.0 indicates the amount of force, that is, equivalent to one’s own body weight.

#### 2.4.2 Passive muscle stiffness

Muscle stiffness was measured in a relaxed state when the participants were lying in a prone position on an examination table (Figure 3B). The passive stiffness of the longissimus muscles was measured using a hand-held myotonometry device (MyotonPRO, Myoton AS, Tallinn, Estonia). This device has been used to monitor changes in muscle stiffness after strenuous exercise (Kong et al., 2018) and to assess the stiffness of previously-injured and uninjured muscles (Nin et al., 2021). The sites of measurement were 2 cm lateral to the L1 spinous process on both left and right sides (Criswell, 2010). The myotonometry device applies a brief mechanical impulse to elicit damped oscillations of the muscle to calculate muscle stiffness (in N/m) (Kong et al., 2018). The average of 5 consecutive measurements was taken on each side.

#### 2.4.3 Muscle fatigability

The modified Sorensen test was used to assess the lumbar extensor muscles’ fatigability using electromyography (EMG) measurements (Cai and Kong, 2015; Cai et al., 2017). In this test, participants were required to lie on an examining table in a prone position with the upper edge of the iliac crests aligned with the edge of the table and the lower body is being strapped down around the pelvis, knees, and ankles (Figure 3C). Participants were asked to maintain a horizontal position for 2 min while keeping their arms folded across the chest. The test would be terminated if participants failed to maintain the upper body in a horizontal position. During the 2-minute test duration, the lumbar extensors muscles activation (longissimus of both left and right sides) was captured using surface EMG (Biomonitor ME6000, Mega Electronics Ltd., Finland). The electrodes were placed on the same measurement sites as the muscle stiffness tests which were 2 cm lateral to the L1 spinous process of each side (Criswell, 2010). Raw EMG data were band-pass filtered at 20–450 Hz and then analyzed in the frequency domain. To examine the back muscle’s resistance to fatigue, the median frequency slope (MFS) of EMG signals was calculated from the power density spectrum (Cai et al., 2017). As muscle fatigue develops over the 2-minute period, the EMG-MFS would decline over time (i.e., negative slope). A steeper slope indicates less resistance to fatigue.

#### 2.4.4 Self-reported disability survey

Perceived back pain and disabilities were assessed using the revised Oswestry Low Back Pain Questionnaire (Fairbank and Pynsent, 2000). This survey asks participants about their back pain intensity during daily situations such as lifting heavy weights, walking, standing, sitting, sleeping, socializing, and the change in degree of pain. There are a total of 10 questions with 6 different options. For each question, the total possible score is 5 (if the first option is marked, score = 0; if the last option is marked, score = 5). The Oswestry Disability Index (ODI) was calculated as a disability score by summing up the scores of all 10 questions. The ODI was then expressed as percentage (0–100%), with higher disability scores indicating greater functional limitation. The classification of the scores was as follow: 0–20%: *minimum functional limitation*; 21–40%: *moderate functional limitation*; 41–60%: *intense functional limitation*; 61–80%: *disability*, and above 80%: *maximum functional limitation* (Fairbank and Pynsent, 2000; Marín-Jiménez et al., 2019).

### 2.5 Statistical analyses

There were missing data among the 24 participants at post-intervention and subsequent follow-up tests due to factors such as loss of interest, work commitment, Ramadan fasting, health, COVID-19, or lost to contact. Missing data were imputed following the last observation carried forward approach. Data were imported into JASP (version 0.14.1) statistical software for analyses, with significance level set at *p* < 0.05. Data are expressed as mean (standard deviation). The outcome variables were strength, stiffness (average of left and right sides), EMG-MFS (average of left and right sides) and ODI. In order to examine the balance between the left and right sides, a symmetry index (%) was calculated for stiffness and EMG-MFS using the formula below (Kong et al., 2010):

$$\mathrm{Symmetry}\;\mathrm{index}=\frac{\left|x_{R}-x_{L}\right|}{0.5\;\left(x_{R}+x_{L}\right)}\times 100\text{\%}$$

where x_R_ represents the variable of the right side and x_L_ represents the variable of the left side.

A mixed model Analysis of Variance (ANOVA) with repeated measures was performed to compare the difference over time and between groups for each variable of interest (*α* = 0.05). Effect size was calculated as partial eta-squared (η_p_^2^). *Post-hoc* tests with Bonferroni adjustments were performed where appropriate.

## 3 Results

Compliance with exercise program was 17.4 (5.5) out of 32 prescribed training sessions over 16 weeks. There was no difference between the Functional (19.3 (7.2)) and Conventional (16.2 (4.1)) group compliance (*p* = 0.184, *d* = 0.578). There was no adverse incident or injuries happened during the supervised training sessions.

### 3.1 Normalized back extension strength

**FIGURE 4:**
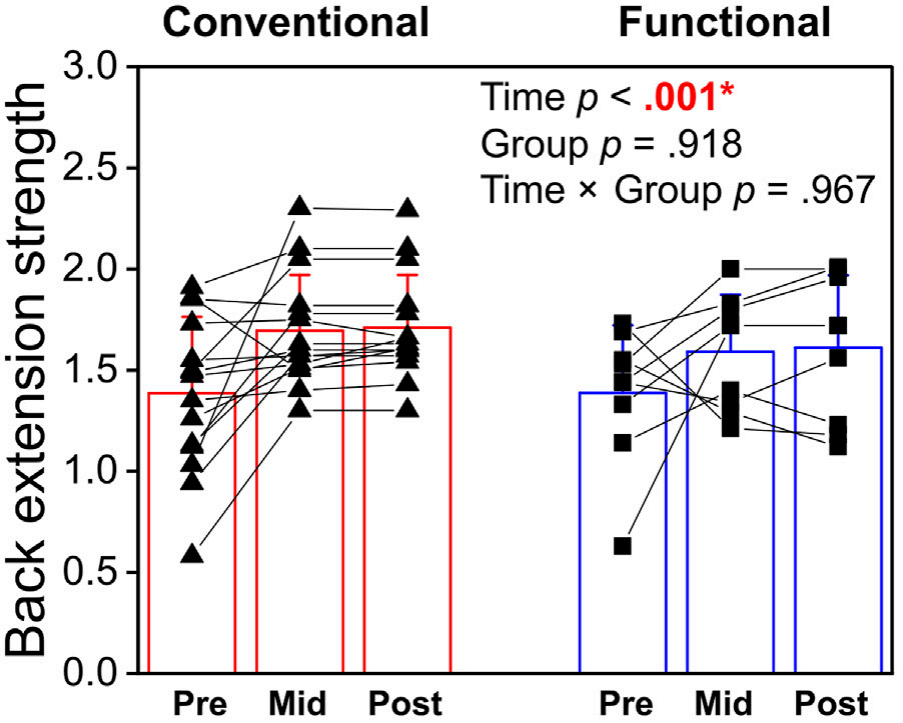
Comparison of the normalized isometric back extension strength between the conventional and functional training at pre-, mid- and post-intervention. *Statistical significance (*p* <0 .05) is shown in red font and indicated by an asterisk.

There was a significant main effect of Time (*p* < 0.001, η_p_^2^ = 0.625, Figure 4) in isometric back extension strength but no interaction effect (*p* = 0.967, η_p_^2^ = 0.002) or difference between the Functional and Conventional groups (*p* = 0.918, η_p_^2^ < 0.001). *Post-hoc* tests revealed that participants increased their back extension strength by 19.6% (+20.3% functional, +18.9% conventional) at mid-intervention (*p* < 0.001) and 20.8% (+21.3% functional, +20.3% conventional) at post-intervention (*p* < 0.001) when compared with baseline (Figure 4). No difference in normalized strength was found between the mid- and post-intervention tests.

### 3.2 Passive muscle stiffness

**FIGURE 5:**
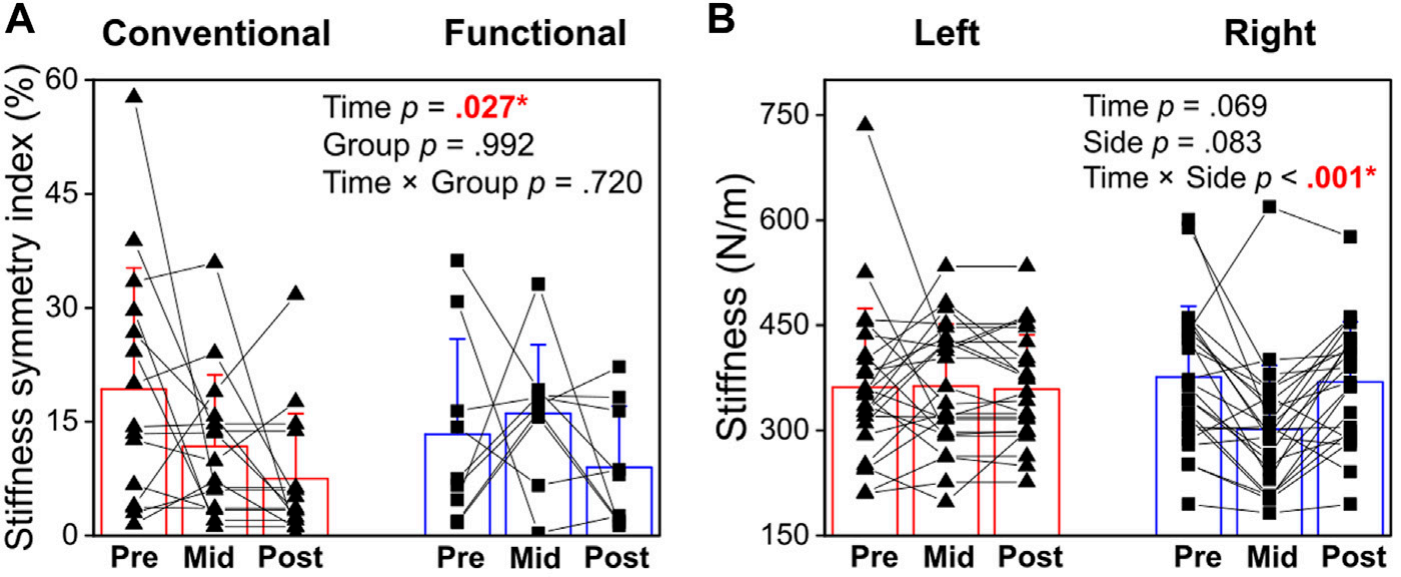
**(A)** Symmetry index of passive muscle stiffness at pre-, mid- and post-intervention in Conventional and Functional training groups. **(B)** Changes in back muscle stiffness over time in left and right back muscles. *Statistical significance (*p* <0 .05) is shown in red font and indicated by an asterisk.

Taking the average of left and right sides, there was no significant effects of Time (*p* = 0.056, η_p_^2^ = 0.123, Figure 5A), Group (*p* =0 .261, η_p_^2^ = 0.057), or Time × Group interaction (*p* =0 .975, η^2^_p_ = 0.011) on back muscle stiffness. The symmetry index of stiffness decreased over time (*p* = 0.027, η_p_^2^ = 0.151), with lower stiffness at post-intervention (-7.1% functional, -11.8% conventional, *p* = 0.023) compared with pre-intervention (Figure 5A). No Group (*p* = 0.992, η_p_^2^ < 0.001) or interaction (*p* = 0.720, η_p_^2^ = 0.015) effects were noted in the stiffness symmetry.

To further understand the left-right balance in muscle stiffness, an additional two-way repeated measures ANOVA was run to compare the stiffness between the left and right sides over time (Figure 5B). The results revealed significant Time × Side interaction (*p* < 0.001 η_p_^2^ = 0.388). At baseline, the back muscle stiffness in the right side was lower than that of the left side (*p* < 0.001) but this left-right imbalance no longer existed in the mid- or post-intervention. No significant main effects of Time (*p* = 0.069, η_p_^2^ = 0.125) or Side (*p* = 0.083, η_p_^2^ = 0.125) were identified. These results showed that left-right symmetry in back muscle stiffness improved after 8 weeks of training, and that this improvement can be maintained with regular exercise training.

### 3.3 Muscle fatigability

**FIGURE 6:**
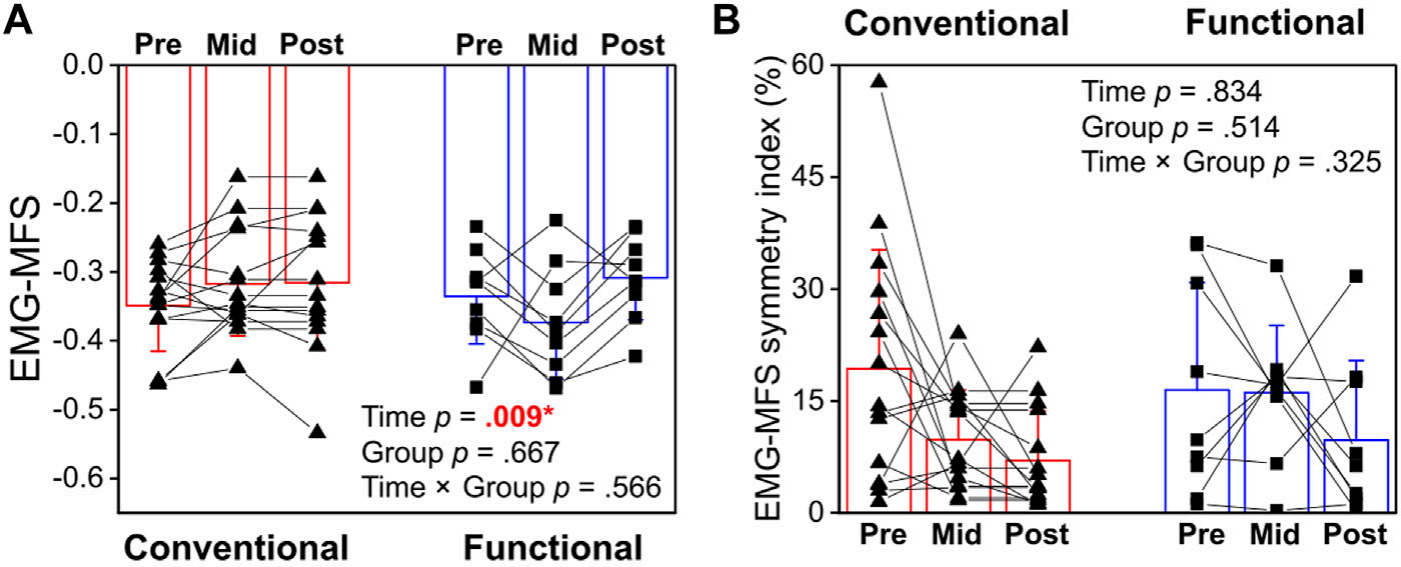
**(A)** Comparison of electromyography median frequency slope (EMG-MFS) between the Conventional and Functional groups at pre-, mid- and post-intervention. **(B)** Changes in symmetry index of EMG-MFS over time between conventional and functional training. *Statistical significance (*p* <0 .05) is shown in red font and indicated by an asterisk.

Taking the average EMG-MFS of the left and right longissimus, there was a significant main effect of Time (*p* = 0.009, η_p_^2^ = 0.191) but not interaction effect (*p* = 0.566, η_p_^2^ = 0.026) or difference between groups (*p* = 0.667, η_p_^2^ = 0.009). A less negative slope indicates improvement in resistance to fatigue (Figure 6A). *Post-hoc* analysis revealed significant 13.6% (+17.4% functional, +9.5% conventional) improvement in fatigability at post-intervention (*p* = 0.009) compared with baseline (Figure 6A). When the symmetry index of EMG-MFS was analyzed, there was no significant effects of Time (*p* = 0.834, η_p_^2^ = 0.008, Figure 6B), Group (*p* = 0.514, η_p_^2^ = 0.020), or Time × Group interaction (*p* = 0.325, η_p_^2^ = 0.050).

### 3.4 Self-reported pain and disability

There was no adverse incident during the training period that caused low back pain or negatively impacted back function. The ODI for lower back pain and functionality are tabulated in Table 3. All disability scores throughout the study period were very low and fell well within the category of “*minimum functional limitation*” (0–20%). There were no significant changes over time (*p* = 0.951, η_p_^2^ = 0.010) or difference between groups (*p* = 0.785, η_p_^2^ = 0.03). While a significant interaction effect was found (*p* = 0.008, η_p_^2^ = 0.131), no pairwise differences could be identified from *post-hoc* analysis.

**TABLE 3:** Self-report Pain and Disability Scores (%) measured using the Oswestry Disability Index (ODI).

	All	Functional Group	Conventional Group
Pre-Intervention	4.8 (14.4)	1.1 (2.0)	7.1 (18.0)
Mid-Intervention	4.8 (14.4)	1.1 (2.0)	7.1 (18.0)
Post-Intervention	4.3 (4.9)	7.3 (10.8)	2.4 (5.0)
2-month Follow-up	4.3 (7.8)	6.9 (8.1)	2.8 (7.4)
4-month Follow-up	3.4 (5.4)	6.2 (5.9)	1.7 (4.5)
6-month Follow-up	2.5 (6.6)	4.9 (9.6)	1.1 (3.6)

*All ODI scores were classified in the category of “Minimal functional limitation” (0–20%). Data are expressed as mean (standard deviation).

## 4 Discussion

This study compared the effectiveness of two strength and conditioning programs, namely functional versus conventional training, that were implemented over 16 weeks in emergency responders. The results did not support our hypothesis that functional training would be superior to conventional training in improving back muscle characteristics and disabilities as there was no difference between groups in any outcome measures. Regardless of the type of exercises prescribed, participants showed improvement in hip extension strength and lumbar extensor fatigability after completing the intervention. Initial left-right imbalance in back muscle stiffness was observed at baseline and the symmetry improved with training. Self-reported pain and disability scores remained low throughout the intervention and 6-month follow-up periods, indicting the high functional ability of the participants.

### 4.1 Improvement in strength and fatigability

The present study showed that back extension strength improved sharply after 8 weeks of training, but no further gain was observed from mid- to post-intervention tests. These results are in line with the literature that 6–8 weeks of exercise training can induce substantial improvement in strength (Moon et al., 2015; Schoenfeld et al., 2015; Steele et al., 2015; Haun et al., 2019; Tay et al., 2019). Given the relatively short duration of 8 weeks, it is believed that most of the initial strength improvement are of a neurological nature rather than muscle hypertrophy (Balshaw et al., 2017). In recreationally trained males, Steele et al. (2015) reported that back extension strength improved after 6 weeks of training with no difference between single-set (8.3% improvement) and multiple-set (10.7% improvement). The current study on emergency responders showed 20.7% of improvement after 8 weeks of training and this initial gain in strength was maintained for a further 8 weeks with regular physical training. The lack of difference between the Functional and Conventional Groups indicates that both types of training can elicit significant increases back muscle extension strength. In the general population, individuals with chronic LBP demonstrated lower strength in lumbar extension and flexion compared with healthy controls (Vanhauter et al., 2021). Thus, regular resistance training is recommended for emergency responders to improve and maintain their back muscles strength.

Lumbar extensor muscle fatigability improved after 16 weeks of training with no difference between functional or conventional exercises. There are currently no data in the available literature regarding the fatigability of back muscles in emergency responders and hence no direct comparison can be made. Previous studies have reported deficit in lumbar extensor endurance among individuals with chronic LBP compared with healthy controls (Klein et al., 1991; Ashmen et al., 1996). Using isoinertial exercise intervention, researchers reported no change in muscle fatigability after a 12-week lumbar extensor training program (Mannion et al., 2001). For runners with chronic LBP, one study demonstrated improvement in longissimus fatigability after 8 weeks of rehabilitation exercise training (Cai et al., 2017). The authors cautioned that despite statistical differences in EMG-MFS were found, the improvement of 0.046 was too small to overcome the minimal detectable changes (MDC, 95% CI) ranging from 0.11 to 0.17. Similar to the study by Cai and colleagues (2017), the present study also observed statistically significant but small improvement in lumbar extensor muscles EMG-MFS after 16 weeks of training (mean change on 0.049 from pre- to post-intervention). Future studies can further investigate the practical relevance of this small improvement in back muscle fatigability and search for optimal strength and conditioning programs for back injuries prevention in emergency responders.

The lack of difference between functional and conventional training may be due to advantage of multi-joint exercises (e.g., squats, push-ups) that were included in both groups, which may have overshadowed other subtle differences (Iversen et al., 2021). In elite soccer players, Turna and Alp (2020) also found no difference between functional and traditional training in biomotor abilities and physiological characteristics. Another point to note is that the outcome assessments of back extension and fatigability were both performed in a bilaterally symmetrical manner. These tests may not fully capture the possible benefits gained via functional training. It will be of interest to explore if the Functional Group demonstrate any advantage in operational-specific movement tasks in the future. Functional outcome assessment tests can be developed to cater for the occupational needs of emergency responders.

### 4.2 Left-right symmetry in muscle stiffness

Stiffness of a muscle unit can influence force production (Kalkhoven and Watsford, 2018; Ando and Suzuki, 2019) and injury risk (Bradshaw and Hume, 2012; Pickering Rodriguez et al., 2017; Nin et al., 2021). At baseline, the passive stiffness of the lumbar extensor muscle of the emergency responders was higher in the left side than the right side (Figure 5B). Koppenhaver et al. (2020) reported that lumbar muscle stiffness was associated with self-reported pain and disability, with greater resting stiffness in individuals with LBP than asymptomatic control. In the literature, an optimal range of back muscle stiffness is yet to be established. An increase in muscle stiffness can contribute to more force production (Kalkhoven and Watsford, 2018; Ando and Suzuki, 2019) but previously injured muscles are found to be stiffer than the non-injured muscles (Nin et al., 2021). On the other hand, muscles with too low stiffness and are too compliant may be more prone to soft-tissue related injuries (Bradshaw and Hume, 2012; Pickering Rodriguez et al., 2017). Most participants (over 85%) in the present study were right-handed. Their limb preference (e.g., the arm operating an axe, the way carrying casualties) may be related to the lower stiffness in the right back muscles. Variations of muscle recruitment patterns could be caused by handedness or training (Merletti et al., 1994; Renkawitz et al., 2006). In a study on tennis players with and without LBP, Renkawitz et al. (2006) discovered that almost all right-handed tennis players showed substantially lower muscle activity on the left back muscles while left-handed players showed lower muscle activity on the right side. For individual with LBP, it is conceivable that they execute different movements between the left and right sides to avoid pain (Shamsi et al., 2020).

There are some evidence to suggest that asymmetry in back muscle properties may lead to back pain or disabilities. Parkhurst and Burnett (1994) found that proprioceptive asymmetries were associated with injuries in male firefighters. In older adults, asymmetrical biomechanical properties of paravertebral muscles are also linked to chronic LBP severity (Wu et al., 2022). The present study provided empirical data to illustrate bilateral asymmetry in back muscle stiffness among emergency responders. As the participants engaged in the supervised strength and conditioning intervention programs, the symmetry index of stiffness progressively decreased over time (Figure 5A). The initial left-right imbalance in muscle stiffness observed at baseline no longer existed in the mid- or post-intervention (Figure 5B). While the two programs emphasized either unilateral or bilaterally loaded exercises, both programs loaded the same muscle groups with equal sets and repetitions and therefore the total training volume was similar. The lack of difference in the programs may indicate that engaging in any well-rounded strength and conditioning program is effective for improving back strength and symmetry and superior to the lack of strength training being performed prior to enrolling in the study.

### 4.3 Self-reported pain and disabilities

The ODI scores were very low among the participants throughout the intervention and 6-month follow-up periods (Table 3). This is expected as our participants were mostly active, frontline firefighters who were physically fit and healthy to perform duties. Since they did not suffer from intense pain or severe functional limitation in the beginning, no improvement in disability measures would be expected. In a study on Iranian EMS personnel, the ODI has decreased from 34.1 to 27.5% (after 1 month) and 19.7% (after 3 months) with ergonomic intervention of patient transfer technique (Yahyaei et al., 2019). In another cross-sectional study on 61 Polish firefighters, the mean ODI was reported as 13.7% and this disability score was not correlated with age (Fiodorenko-Dumas et al., 2018). Compared with the ODI values of emergency responders reported in other countries (13.7–34.1%), the emergency responders in Singapore who participated in our study reported less pain and disabilities associated with their back throughout the entire study duration (mean 2.5–4.8%, Table 3).

### 4.4 Limitations

There are some limitations to the present study. First, the study plan was severely interrupted by COVID-19 especially during the early stage of the training. The frequent changes and strict restrictions (e.g., closure of gym, group size limitation), coupling with change in work arrangement, resulted in a high drop-out rate in the first few weeks. Given the relatively small sample size of 24 participants, future work on a larger cohort is warranted to confirm the present findings. Second, the compliance rate for supervised training was lower than expected. Out of the 32 planned training sessions, participants only attended 17 (ranging from 9 to 30) sessions on average. On-duty strength training has been promoted as one way to improve health and fitness among emergency responders, with promising positive impact on injury prevention (Griffin et al., 2016). Despite the present study conducted training of the participants’ duty days, the compliance rate remained low. This highlighted the challenges of implementing physical training programs in frontline emergency responders who have different work shifts and other priorities. Third, the post-intervention test was conducted around the Ramadan fasting period during which the exercise and diet routines of our Muslim participants were affected. Some participants missed training towards the end. Others delayed the post-intervention tests until after the fasting period. As such, the training effect may not be accurately captured by the post-intervention test results.

## 5 Conclusion

This study showed that 16 weeks of strength and conditioning training was promising in improving back extension strength, bilateral symmetry in back muscle stiffness, and lumbar extensor muscle fatigability. The training effects were similar between functional exercises (which puts more emphasis on unilateral movements) and conventional exercises (which tends to be more bilateral symmetrical). All self-reported pain and disability scores were very low and fell well within the category of “*minimum functional limitation*” throughout the study. Therefore, we conclude that a structured strength training program targeting all major muscle groups improves back strength, stiffness symmetry and fatigability among firefighters and paramedics. Functional training does not provide superior results to conventional strength training in this interval and cohort of firefighters and paramedics. Future studies can examine if long-term compliance to strength and conditioning programs can improve occupational performance and/or to reduce the risk of injuries in emergency responders.

## Data Availability

The datasets presented in this study can be found in online repositories. The names of the repository/repositories and accession number(s) can be found below: The datasets generated for this study can be found in the NIE Data Repository [https://doi.org/10.25340/R4/CLHRWV].
