# Supplementary material for: Functional versus conventional strength and conditioning programs for back injury prevention in emergency responders
Source: Front Bioeng Biotechnol. 2022 Sep 9;10:918315. doi: 10.3389/fbioe.2022.918315 (PMC9500301; doi:10.3389/fbioe.2022.918315)
Supplement: Supplementary file 1 [file Table1.DOCX]

Supplementary Material

# Supplementary Table 1. Functional Training Program for 16 Weeks

| **Set** | **Week 1 Day 1** | **Week 1 Day 2** | **Week 2 Day 1** | **Week 2 Day 2** |
| --- | --- | --- | --- | --- |
| A1 | Reverse Lunges | Box Step Up | Walking Lunges | Single Leg Kettlebell Deadlift |
| A2 | Dumbbell Bench Press | Incline Dumbbell Bench Press | Seesaw Bench Press | Plank to pillar |
| B1 | Single Leg Kettlebell Deadlift | Half Kneeling Single Arm Press | Russian Kettlebell Swing | Seesaw Overhead Press |
| B2 | Bent Over Kettlebell Row | Bent Over Kettlebell Row | Renegade Row | Standing Cable Lift |
| C1 | Forearm Plank | Hollow Body Hold | Crab Walk (15 m) | Kettlebell Flutter Kick |
| C2 | Single Arm Upright Row | Plank Hold Dumbbell Drag | Standing Cable Chop | Standing Double Arm Face Pull |
| D | Tabata | EMOM^1^ (6 min) | Tabata | EMOM (9 min) |
|  | Burpees | 100-m treadmill run | Push-ups | Pull-ups |
|  | Air Squats | Bear Crawl Hold | V-ups | Sit-ups |
|  |  |  |  | Air Squats |
| **Set** | **Week 3 Day 1** | **Week 3 Day 2** | **Week 4 Day 1** | **Week 4 Day 2** |
| A1 | Reverse Lunges | Single Leg Kettlebell Deadlift | Goblet Cossack Squat | Single Leg Kettlebell Deadlift |
| A2 | Single Arm Bench Press | Plank Walkouts | Seesaw Bench Press | Spiderman Crawl |
| B1 | Russian Kettlebell Swing | Seesaw Overhead Press | Russian Kettlebell Swing | Single Arm Dumbbell Press |
| B2 | Bent Over Seesaw Row | Standing Cable Lift | Bent Over Kettlebell Row | Standing Cable Lift |
| C1 | Reverse Quadruped Crawl | Kettlebell Deadbug | Bear Crawl + Drag | Kettlebell Deadbug |
| C2 | Standing Cable Chop | Weighted Reverse Snow Angels | Standing Cable Chop | Trap-3 Raise |
| D | Tabata | EMOM (9 min) | Conditioning (6 minutes) | EMOM (6 min) |
|  | Push-ups | Step-ups | Pull-ups | 30-sec Kettlebell Swings |
|  | Flutter Kicks | Burpees | Push ups | 30-sec Burpees |
|  |  | Sit-ups | Air Squats |  |
| **Set** | **Week 5 Day 1** | **Week 5 Day 2** | **Week 6 Day 1** | **Week 6 Day 2** |
| A1 | Reverse Lunges | Box Step Up | Dumbbell Reverse Lunges | Single Leg Kettlebell Deadlift |
| A2 | Dumbbell Bench Press | Incline Dumbbell Bench Press | Seesaw Bench Press | Plank to Pillar |
| B1 | Single Leg Kettlebell Deadlift | Half Kneeling Single Arm Press | Russian Kettlebell Swing | Seesaw Overhead Press |
| B2 | Bent Over Kettlebell Row | Bent Over Kettlebell Row | Renegade Row | Standing Cable Lift |
| C1 | Forearm Plank | Hollow Body Hold | Lateral Squats | Kettlebell Flutter Kick |
| C2 | Single Arm Upright Row | Plank Hold Dumbbell Drag | Standing Cable Chop | Standing Double Arm Face Pull |
| D | Tabata | EMOM (6 min) | Tabata | EMOM (9 min) |
|  | Burpees | 100-m treadmill run | V-ups | Pull-ups |
|  | Squats | Bear Crawl Hold | Push-ups | Sit-ups |
|  |  |  | Air Squats | Air Squats |
| **Set** | **Week 7 Day 1** | **Week 7 Day 2** | **Week 8 Day 1** | **Week 8 Day 2** |
| A1 | Reverse Lunges | Single Leg Kettlebell Deadlift | Goblet Cossack Squat | Single Leg Kettlebell Deadlift |
| A2 | Single Arm Bench Press | Plank Walkouts | Seesaw Bench Press | Spiderman Crawl |
| B1 | Russian Kettlebell Swing | Seesaw Overhead Press | Russian Kettlebell Swing | Single Arm Dumbbell Press |
| B2 | Bent Over Seesaw Row | Standing Cable Lift | Bent Over Kettlebell Row | Standing Cable Lift |
| C1 | Reverse Quadruped Crawl | Kettlebell Deadbug | Bear Crawl + Drag | Kettlebell Deadbug |
| C2 | Standing Cable Chop | Weighted Reverse Snow Angels | Standing Cable Chop | Trap-3 Raise |
| D | Tabata | EMOM (9 min) | Conditioning (6 min) | EMOM (6 min) |
|  | Push-ups | Step-ups | Pull-ups | 30-sec Kettlebell Swings |
|  | Flutter Kicks | Burpees | Push-ups | 30-sec Burpees |
| **Set** | **Week 9 Day 1** | **Week 9 Day 2** | **Week 10 Day 1** | **Week 10 Day 2** |
| A1 | Reverse Lunges | Dumbbell Box Step Up | Walking Lunges | Single Leg Kettlebell Deadlift |
| A2 | Dumbbell Bench Press | Incline Dumbbell Bench Press | Seesaw Bench Press | Plank to pillar |
| B1 | Single Leg Kettlebell Deadlift | Half Kneeling Single Arm Press | Russian Kettlebell Swing | Seesaw Overhead Press |
| B2 | Bent Over Kettlebell Row | Bent Over Kettlebell Row | Renegade Row | Standing Cable Lift |
| C1 | Forearm Plank | Hollow Body Hold | Crab Walk | Kettlebell Flutter Kick |
| C2 | Single Arm Upright Row | Plank Hold Dumbbell Drag | Standing Cable Chop | Standing Double Arm Face Pull |
| D | Tabata | EMOM^1^ (6 min) | Conditioning (6 min) | EMOM (9 min) |
|  | Burpees | 100-m treadmill sprint | V-ups | Pull-ups |
|  | Air Squats | Bear Crawl Hold | Push-ups | Sit-ups |
|  |  |  |  | Air Squats |
| **Set** | **Week 11 Day 1** | **Week 11 Day 2** | **Week 12 Day 1** | **Week 12 Day 2** |
| A1 | Reverse Lunges | Single Leg Kettlebell Deadlift | Goblet Cossack Squat | Single Leg Kettlebell Deadlift |
| A2 | Single Arm Bench Press | Plank Walkouts | Seesaw Bench Press | Spiderman Crawl |
| B1 | Russian Kettlebell Swing | Seesaw Overhead Press | Russian Kettlebell Swing | Single Arm Dumbbell Press |
| B2 | Bent Over Seesaw Row | Standing Cable Lift | Bent Over Kettlebell Row | Standing Cable Lift |
| C1 | Reverse Quadruped Crawl | Kettlebell Deadbug | Bear Crawl + Drag | Kettlebell Deadbug |
| C2 | Standing Cable Chop | Weighted Reverse Snow Angels | Standing Cable Chop | Trap-3 Raise |
| D | Tabata | EMOM (9 min) | Conditioning (6 min) | EMOM (6 min) |
|  | Push-Ups | Step ups | Pull-ups | 30-sec Kettlebell Swings |
|  | Flutter Kicks | Burpees | Push-ups | Burpees |
|  |  | Sit-ups | Air Squats |  |
| **Set** | **Week 13 Day 1** | **Week 13 Day 2** | **Week 14 Day 1** | **Week 14 Day 2** |
| A1 | Reverse Lunges | Box Step Up | Walking Lunges | Single Leg Kettlebell Deadlift |
| A2 | Dumbbell Bench Press | Incline Dumbbell Bench Press | Seesaw Bench Press | Plank to Pillar |
| B1 | Single Leg Kettlebell Deadlift | Half Kneeling Single Arm Press | Russian Kettlebell Swing | Seesaw Overhead Press |
| B2 | Bent Over Kettlebell Row | Bent Over Kettlebell Row | Renegade Row | Standing Cable Lift |
| C1 | Forearm Plank | Hollow Body Hold | Crab Walk | Kettlebell Flutter Kick |
| C2 | Single Arm Upright Row | Plank Hold Dumbbell Drag | Standing Cable Chop | Standing Double Arm Face Pull |
| D | Tabata | EMOM (6 min) | Tabata | EMOM (9 min) |
|  | Burpees | 100-m treadmill Sprint | Push-ups | Pull-ups |
|  | Air Squats | Bear Crawl Hold | V-ups | Sit-ups |
|  |  |  |  | Squats |
| **Set** | **Week 15 Day 1** | **Week 15 Day 2** | **Week 16 Day 1** | **Week 16 Day 2** |
| A1 | Reverse Lunges | Single Leg Kettlebell Deadlift | Goblet Cossack Squat | Single Leg Kettlebell Deadlift |
| A2 | Single Arm Bench Press | Plank Walkouts | Seesaw Bench Press | Spiderman Crawl |
| B1 | Russian Kettlebell Swing | Seesaw Overhead Press | Russian Kettlebell Swing | Single Arm Dumbbell Press |
| B2 | Bent Over Seesaw Row | Standing Cable Lift | Bent Over Kettlebell Row | Standing Cable Lift |
| C1 | Reverse Quadruped Crawl | Kettlebell Deadbug | Bear Crawl + Dumbbell Drag | Kettlebell Deadbug |
| C2 | Standing Cable Chop | Weighted Reverse Snow Angels | Standing Cable Chop | Trap-3 Raise |
| D | Tabata | EMOM (9 min) | Conditioning (6 min) | EMOM (6 min) |
|  | Push-ups | Step-ups | Pull-ups | Kettlebell Swings |
|  | Flutter Kicks | Burpees | Push-ups | Burpees |
|  |  | Sit-ups | Air Squats |  |

^1^EMOM – every minute on the minute (completing an exercise within one minute)
