# Supplementary material for: Functional versus conventional strength and conditioning programs for back injury prevention in emergency responders
Source: Front Bioeng Biotechnol. 2022 Sep 9;10:918315. doi: 10.3389/fbioe.2022.918315 (PMC9500301; doi:10.3389/fbioe.2022.918315)
Supplement: Supplementary file 2 [file Table2.DOCX]

Supplementary Material

# Supplementary Table 2. Conventional Training Program for 16 Weeks

| **Set** | **Week 1 Day 1** | **Week 1 Day 2** | **Week 2 Day 1** | **Week 2 Day 2** |
| --- | --- | --- | --- | --- |
| A1 | Back Squat | Back Squat | Back Squat | Sumo Deadlift |
| A2 | Push-ups | Bench Press | Plank Hold | Hanging Knee Raise |
| B1 | Romanian Deadlift | Strict Press | Barbell Good Morning | Pause Bench Press |
| B2 | Bent Over Barbell Row | Seal Row | Dumbbell Fly | Pull-ups |
| C1 | Forearm Plank | Hollow Body Hold | Side Plank | Strict Press |
| C2 | Bilateral Upright Row | Lats Pull Down | Lats Pull Down | Bent Over Barbell Row |
| D | Treadmill HIIT^1^ (4 sets) | Cycle HIIT (6 sets) | Cycle HIIT (8 sets) | Treadmill HIIT (9 sets) |
|  | 20-sec Sprint | 20-sec Maximum Effort | 20-sec Maximum Effort | 20-sec Sprint |
|  | 40-sec Rest | 40-sec Rest | 10-sec Rest | 40-sec Rest |
| **Set** | **Week 3 Day 1** | **Week 3 Day 2** | **Week 4 Day 1** | **Week 4 Day 2** |
| A1 | Back Squat | Sumo Deadlift | Back Squat | Sumo Deadlift |
| A2 | Quadruped Hold | Hollow Body Hold | Weighted Plank | Sit-ups |
| B1 | Romanian Deadlift | Bench Press | Body Row | Bench Press |
| B2 | Push-ups | Pull-ups | Bench Dips | Lats Pull Down |
| C1 | Side Plank | Strict Press | Side Plank | Strict Press |
| C2 | Lats Pull Down | Seated Cable Row | Pull-ups | Pendlay Row |
| D | Cycle HIIT (8 sets) | Treadmill HIIT (9 sets) | Cycle HIIT (6 sets) | Treadmill HIIT (6 sets) |
|  | 20-sec Maximum Effort | 20-sec Sprint | 30-sec Maximum Effort | 30-sec Sprint |
|  | 10-sec Rest | 40-sec Rest | 10-sec Rest | 30-sec Rest |
| **Set** | **Week 5 Day 1** | **Week 5 Day 2** | **Week 6 Day 1** | **Week 6 Day 2** |
| A1 | Back Squat | Back Squat | Back Squat | Sumo Deadlift |
| A2 | Push-ups | Bench Press | Plank Hold | Hanging Knee Raise |
| B1 | Romanian Deadlift | Strict Press | Barbell Good Morning | Pause Bench Press |
| B2 | Bent Over Barbell Row | Bent Over Barbell Row | Dumbbell Shoulder Fly | Pull-ups |
| C1 | Forearm Plank | Hollow Body Hold | Side Plank | Strict Press |
| C2 | Bilateral Upright Row | Lats Pull Down | Lats Pull Down | Bent Over Barbell Row |
| D | Treadmill HIIT (8 sets) | Cycle HIIT (6 sets) | Cycle HIIT (8 sets) | Treadmill HIIT (9 sets) |
|  | 20-sec Sprint | 20-sec Maximum Effort | 20-sec Maximum Effort | 20-sec Sprint |
|  | 40-sec Rest | 40-sec Rest | 10-sec Rest | 40-sec Rest |
| **Set** | **Week 7 Day 1** | **Week 7 Day 2** | **Week 8 Day 1** | **Week 8 Day 2** |
| A1 | Back Squat | Sumo Deadlift | Back Squat | Sumo Deadlift |
| A2 | Quadruped Hold | Hollow Body Hold | Weighted Plank | Sit-ups |
| B1 | Romanian Deadlift | Bench Press | Body Row | Bench Press |
| B2 | Push-ups | Pull-Up | Bench Dips | Lats Pull Down |
| C1 | Side Plank | Strict Press | Side Plank | Strict Press |
| C2 | Lats Pull Down | Seated Cable Row | Pull-ups | Pendlay Row |
| D | Cycle HIIT (8 sets) | Treadmill HIIT (9 sets) | Cycle HIIT (6 sets) | Treadmill HIIT (6 sets) |
|  | 20-sec Maximum Effort | 20-sec Sprint | 30-sec Maximum Effort | 30-sec Sprint |
|  | 10-sec Rest | 40-sec Rest | 10-sec Rest | 30-sec Rest |
| **Set** | **Week 9 Day 1** | **Week 9 Day 2** | **Week 10 Day 1** | **Week 10 Day 2** |
| A1 | Back Squat | Back Squat | Back Squat | Sumo Deadlift |
| A2 | Push-ups | Bench Press | Plank Hold | Hanging Knee Raise |
| B1 | Romanian Deadlift | Strict Press | Barbell Good Morning | Pause Bench Press |
| B2 | Bent Over Barbell Row | Bent Over Barbell Row | Dumbbell Fly | Pull-ups |
| C1 | Forearm Plank | Hollow Body Hold | Side Plank | Strict Press |
| C2 | Bilateral Upright Row | Lats Pull Down | Lats Pull Down | Bent Over Barbell Row |
| D | Treadmill HIIT^1^ (6 sets) | Cycle HIIT (6 sets) | Cycle HIIT (8 sets) | Treadmill HIIT (9 sets) |
|  | 20-sec Sprint | 20-sec Maximum Effort | 20-sec Maximum Effort | 20-secSprint |
|  | 40-sec Rest | 40-sec Rest | 10-sec Rest | 40-sec Rest |
| **Set** | **Week 11 Day 1** | **Week 11 Day 2** | **Week 12 Day 1** | **Week 12 Day 2** |
| A1 | Back Squat | Sumo Deadlift | Back Squat | Sumo Deadlift |
| A2 | Quadruped Hold | Hollow Body Hold | Weighted Plank | Sit-ups |
| B1 | Romanian Deadlift | Bench Press | Body Row | Bench Press |
| B2 | Push-ups | Pull-ups | Bench Dips | Lats Pull Down |
| C1 | Side Plank | Strict Press | Side Plank | Strict Press |
| C2 | Lats Pull Down | Seated Cable Row | Pull-ups | Pendlay Row |
| D | Cycle HIIT (8 sets) | Treadmill HIIT (9 sets) | Cycle HIIT (6 sets) | Treadmill HIIT (6 sets) |
|  | 20-sec Maximum Effort | 20-sec Sprint | 30-sec Maximum Effort | 30-sec Sprint |
|  | 10-sec Rest | 40-sec Rest | 10-sec Rest | 30-sec Rest |
| **Set** | **Week 13 Day 1** | **Week 13 Day 2** | **Week 14 Day 1** | **Week 14 Day 2** |
| A1 | Back Squat | Back Squat | Back Squat | Sumo Deadlift |
| A2 | Push-ups | Bench Press | Plank Hold | Hanging Knee Raise |
| B1 | Romanian Deadlift | Strict Press | Barbell Good Morning | Pause Bench Press |
| B2 | Bent Over Barbell Row | Bent Over Barbell Row | Dumbbell Shoulder Fly | Pull-ups |
| C1 | Side Plank | Hollow Body Hold | Side Plank | Strict Press |
| C2 | Bilateral Upright Row | Lats Pull Down | Lats Pull Down | Bent Over Barbell Row |
| D | Treadmill HIIT (4 sets) | Cycle HIIT (6 sets) | Cycle HIIT (8 sets) | Treadmill HIIT (9 sets) |
|  | 20-sec Sprint | 20-sec Maximum Effort | 20-sec Maximum Effort | 20-sec Sprint |
|  | 40-sec Rest | 40-sec Rest | 10-sec Rest | 40-sec Rest |
| **Set** | **Week 15 Day 1** | **Week 15 Day 2** | **Week 16 Day 1** | **Week 16 Day 2** |
| A1 | Back Squat | Sumo Deadlift | Back Squat | Sumo Deadlift |
| A2 | Quadruped Hold | Hollow Body Hold | Weighted Plank | Sit-ups |
| B1 | Romanian Deadlift | Bench Press | Body Row | Bench Press |
| B2 | Push-ups | Pull-Up | Bench Dips | Lats Pull Down |
| C1 | Side Plank | Strict Press | Side Plank | Strict Press |
| C2 | Lats Pull Down | Seated Cable Row | Pull-ups | Pendlay Row |
| D | Cycle HIIT (8 sets) | Treadmill HIIT (9 sets) | Cycle HIIT (6 sets) | Treadmill HIIT (6 sets) |
|  | 20-sec Maximum Effort | 20-sec Sprint | 30-sec Maximum Effort | 30-sec Sprint |
|  | 10-sec Rest | 40-sec Rest | 10-sec Rest | 30-sec Rest |

^1^HIIT – high intensity interval training
